# HCV compliance and treatment success rates are higher with DAAs in structured HCV clinics compared to general hepatology clinics

**DOI:** 10.1097/MD.0000000000016242

**Published:** 2019-07-12

**Authors:** Navdeep Chehl, Anurag Maheshwari, Hwan Yoo, Colleen Cook, Talan Zhang, Sara Brown, Paul J. Thuluvath

**Affiliations:** Institute for Digestive Health and Liver Diseases, Mercy Medical Center, Baltimore, MD.

**Keywords:** compliance, structured HCV Clinic, treatment Success

## Abstract

The real-world cure rates for hepatitis C (HCV) with direct-acting antivirals (DAAs) based on intention-to-treat (ITT) analysis may be lower than reported in the literature because of non-compliance.

To determine whether patients treated in a structured outpatient HCV clinic (SHC) had higher compliance and treatment success rates compared to those treated in general hepatology clinics (GHC).

In this study, we compared the treatment and compliance success rates of 488 and 840 patients treated in the SHC and GHC, respectively. The SHC required a pre-treatment clinic visit when patients picked up their initial medication, and received detailed education of the treatment plan and follow-up. In the GHC, the medications were delivered to patients’ homes, and there was less formal education. Compliance success was defined as a combination of treatment completion and obtaining at least 1 post-treatment viral load at week 4 or 12. Treatment success was defined as either SVR4 or SVR12.

Fifty of 488 (10.3%) patients from the SHC and 163 of 840 (19.4%) patients from the GHC were lost to follow-up (*P* < .0001). sustained virological response (SVR) rates were similar in compliant patients in both the SHC (419/438, 95.6%) and GHC (642/677, 94.8%), but treatment success rates by intention to treat (ITT) (overall 79.9%) were higher in SHC compared to GHC (85.9% vs 76.4%, *P* < .0001). Multivariate analysis showed that female patients (*P* = .01), older age (*P* = .0005), treatment in SHC (OR 1.7, 95% CI 1.2, 2.3, *P* = .0008), and sofosbuvir/simeprevir compared to sofosbuvir/ledipasvir had higher odds of compliance success; elbasvir/grazoprevir or dasabuvir/ombitasvir/paritaprevir/ritonavir had lower odds of compliance success compared to sofosbuvir/ledipasvir. Female patients (*P* = .02), older age (*P* < .0001), previous treatment (*P* = .03), treatment in SHC (OR 1.7, 95% CI 1.2, 2.3, *P* = .0008), and sofosbuvir/ledipasvir compared to sofosbuvir/velpatasvir, sofosbuvir, or elbasvir/grazoprevir had higher odds of treatment success. With 1:1 matching, the SHC group still had significantly higher odds than the GHC group of achieving treatment and compliance success.

Our study shows that the effectiveness of HCV treatment could be improved by coordinating treatment in a structured HCV clinic.

## 1 Introduction

Hepatitis C virus (HCV) infection is estimated to affect over 71 million people worldwide.^[1]^ In the United States, approximately 3.5 million people are infected with chronic HCV^[2]^ and HCV causes more than 400,000 deaths per year globally, mostly related to complications associated with cirrhosis.^[3]^ The proportion of cirrhosis in chronically infected patients is rising and projected to reach 44.9% by 2030.^[4]^ The availability of multiple pan-genotypic, interferon-free, oral, direct-acting antiviral (DAA) treatment regimens has drastically altered the landscape of HCV treatment. These DAA regimens are simple, safe, well-tolerated, and highly effective, with reported sustained virologic response (SVR) rates exceeding 95% in patients with compensated liver disease.^[5]^ SVR after HCV-directed therapy is associated with an improvement in HCV-related liver injury, and this may lead to liver fibrosis regression, and a reduction in the incidence of hepatocellular carcinoma (HCC), thereby prolonging overall survival.^[5–9]^

Multiple studies have evaluated the real-world effectiveness of DAAs and have demonstrated SVR rates comparable to those reported in clinical trials. However, many of these studies reported SVR data based on patients who completed treatment (per protocol analysis) and post-treatment HCV RNA testing; patients who did not have post-treatment HCV RNA testing were excluded from these studies.^[10–15]^ However, SVR rates were significantly lower when patients who discontinued treatment or lost to follow-up were included in the analysis (intention-to-treat) of few real-life studies.^[16–19]^ The studies that showed lower SVR showed that a significant proportion of patients treated with DAAs either discontinued treatment or were lost to follow-up.

A recent community-based, non-randomized study showed that there were no differences in SVR rates when treatment was administered by nurse practitioners, primary care physicians or specialists (5 infectious disease specialists and 1 hepatologist).^[17]^ Nevertheless, SVR rates ranged from 75% to 100% among the providers, suggesting a wide variability in SVR rates that could be due to patient factors, clinic setting, or other hitherto unknown variables.^[17]^ We hypothesized that real-world SVR rates based on an intention-to-treat (ITT) analysis (‘effectiveness’) may be lower than reported in the literature because of non-compliance of patients, and moreover, the compliance could be improved if patients were managed in a structured outpatient HCV clinic. The objective of our study was to explore whether patients treated in a structured outpatient HCV clinic (SHC) had higher compliance and treatment success rates compared to those treated in the general hepatology clinic (GHC).

## 2 Materials and methods

This was a retrospective, intention-to-treat cohort study which included patients treated with an interferon-free DAA regimen at Mercy Medical Center in Baltimore, Maryland. All patients who were confirmed to have initiated DAA treatment, starting from the introduction of DAA therapy at Mercy Medical Center, were eligible for the study. We did not include patients who finished therapy after November 30th, 2017. During the study period, the following DAA regimens were used: sofosbuvir, sofosbuvir/daclatasvir, sofosbuvir/ ledipasvir, sofosbuvir/velpatasvir, sofosbuvir/simeprevir, elbasvir/grazoprevir, or dasabuvir/ombitasvir/paritaprevir/ritonavir. Ribavirin was used for some patients with every treatment. The choice of treatment regimen was at the discretion of the provider, based on clinical characteristics, and insurance. The frequency of follow-up or laboratory testing was dependent on care coordination in the SHC vs GHC.

### 2.1 Clinic setting

Patients were either treated by 1 experienced hepatologist in the SHC or by 2 experienced hepatologists in the GHC. All patients seen for initial consultation, regardless of the clinic setting, had baseline viral load testing and fibrosis staging with Fibroscan and/or liver biopsy. In the SHC, patients were required to have a subsequent pre-treatment clinic visit, at which they pick-up the initial medication supply, as well as receive detailed instructions regarding the treatment plan, medication side effects, drug-drug interactions, laboratory monitoring, and follow-up appointments. Medication refills were delivered directly to the patients. Follow-up SHC visits tracked flowsheet documentation of blood tests. A viral load (commercially available HCV RNA, quantitative, real-time PCR obtained through Quest Diagnostics or LabCorp) was obtained during treatment, at the end of treatment, at 4 weeks post-treatment, and at 12 weeks post-treatment. In the GHC, patients had all medications, including the initial supply, delivered directly to their homes. Thus, patients had the option to initiate the medications without review of information and instructions from the provider. The GHC had less formal treatment plans and lacked flowsheet documentation during follow-up visits, and laboratory monitoring and follow-up varied based on the provider. An HCV coordinator was accessible in the SHC and GHC to assist in the coordination of care and answer patient questions.

### 2.2 Clinical outcomes

Achievement of SVR was defined as an “undetectable” viral load (reported either quantitatively below the laboratory's lower limit of quantification or qualitatively as “undetectable”) that was obtained 4 or more weeks post-treatment (SVR4) or 12 or more weeks post-treatment (SVR12). End of therapy (EOT) was determined by the last day of treatment per the patient history as documented in the progress note. “Compliance success” was achieved if a patient completed the treatment and had viral load testing at post-treatment week 4 and/or post-treatment week 12. “Treatment success” was achieved if a patient had negative viral load testing consistent with SVR4 or SVR12.

### 2.3 Statistical analysis

Descriptive statistics for characteristics of patients were presented as means and standard deviations (SDs) for continuous variables, and frequencies for categorical variables. The patients’ characteristics difference between the SHC and GHC was assessed by using Chi-square test for categorical variables, and *T* test for continuous variables; a variable with *P* value ≤.05 indicates a significant difference between 2 groups. Baseline covariates included age, gender, race, marital status, insurance, median income as stratified by zip code (obtained using the United States Census Data for the period of 2006–2010), employment status, drug, and alcohol abstinence for more than 1 year, presence of cirrhosis, history of liver transplant, treatment regimen, treatment duration, use of ribavirin, previous treatment, and treatment setting (SHC or GHC). Logistic regression with ITT approach was used to examine whether these characteristics were separately associated with compliance success and treatment success. An unadjusted model was developed first, followed by an adjusted model, which included any variable with individual (univariate) effects (*P* ≤ .05) on outcomes. The final model retained the variables with *P* value ≤.05.

As a sensitivity analysis, to account for confounding due to clinic setting selection bias, 2-stage propensity score matching was implemented. First, the propensity score was calculated based on logistic regression with clinic setting group as the outcome variable, and age, race, marital status, insurance, household median income, drug, and alcohol abstinence for more than 1 year, presence of cirrhosis, history of liver transplant, post-transplant and treatment regimen as covariates. SHC treated patients were then matched 1:1 to GHC treated patients using propensity scores. Balance of covariates between SHC and GHC treated patients were compared after matching. Logistic regression analysis was performed based on the matched cohort to evaluate if there was a significant difference between the SHC and GHC on compliance success and treatment success separately.

The study was approved by the Institutional Review Board at Mercy Medical Center in Baltimore, MD.

## 3 Results

A total of 1328 patients, 488 patients in the SHC and 840 patients in the GHC, were included in the study. The SHC group had a significantly higher proportion of patients who were white, married, with private insurance, living in zip codes with higher median income, and abstinent from drugs or alcohol. However, the SHC group also had a higher proportion of patients with cirrhosis, previous treatment failures and those who had liver transplantation (Table 1).

**Table 1:**
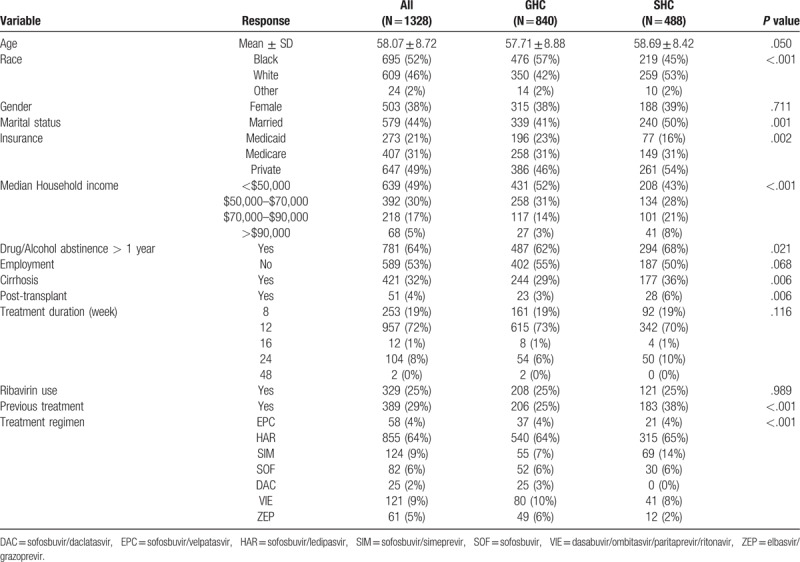
Baseline characteristics stratified by clinic setting.

Overall, 86.7% (1151/1328) of patients completed the treatment. During the study period, 10.3% (50/488) of patients were lost to follow-up with no SVR labs in the SHC, compared with 19.4% (163/840) of patients in the GHC (*P* < .0001). Of the patients who were not lost to follow-up, 95.7% (419/438) of patients achieved SVR in the SHC and 94.8% (642/677) of patients achieved SVR in the GHC (*P* = .5).

By ITT, the overall SVR rate was 79.9%; the treatment success rate was significantly higher in the SHC compared with the GHC (419/488 = 85.9% vs 642/840 = 76.4%, *P* < .0001).

**Figure 1:**
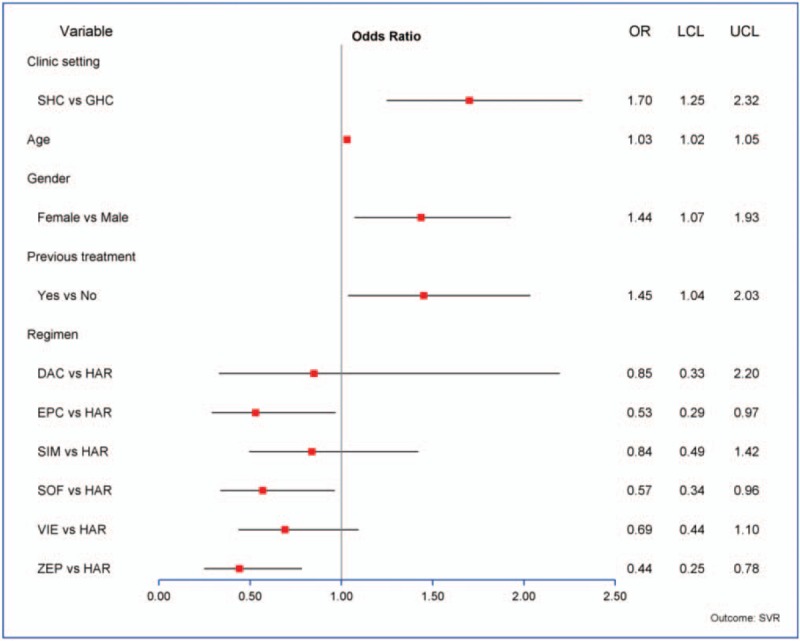
Adjusted odds ratio of treatment success (multivariate model).

Univariate analysis showed other variables individually associated with treatment success included age (*P* < .0001), insurance (*P* = .005), gender (*P* = .05), previous treatment (*P* = .02), and treatment regimen (*P* = .02) (Table 2). In the final multivariate model, clinic setting still showed significant effect after adjustment by age, gender, previous treatment, and treatment regimen. SHC treated patients had higher odds of achieving SVR than GHC treated patients (OR 1.7, 95% CI 1.3, 2.3, *P* = .001). Female patients (OR 1.4, 95% CI: 1.1, 1.9, *P* = .02), older age (OR 1.03, 95% CI: 1.02, 1.05, *P* < .0001), patients who had previous treatment (OR 1.5, 95% CI: 1.04, 2.03, *P* = .031), and patients who had sofosbuvir/ledipasvir compared to sofosbuvir/velpatasvir, sofosbuvir, or elbasvir/grazoprevir had higher odds of treatment success (Fig. 1).

**Table 2:**
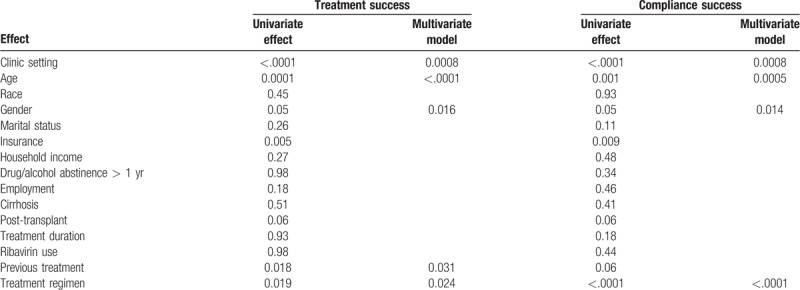
Univariate and multivariate analysis of treatment success and compliance success.

**Figure 2:**
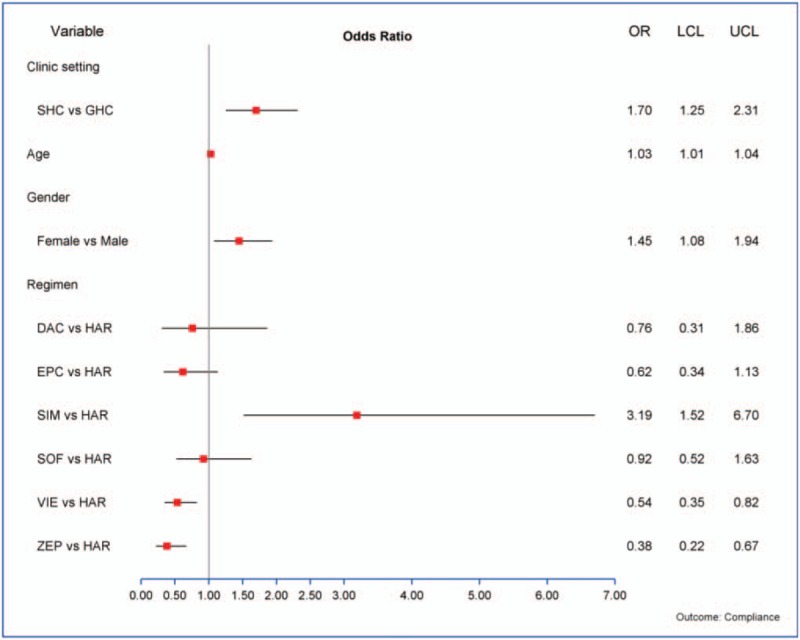
Adjusted odds ratio of compliance success (multivariate model).

The overall compliance success rate was 79.9% (1061/1328). By ITT, the compliance success rate was significantly higher in the SHC compared with the GHC (420/488 = 86.1% vs 641/840 = 76.3%, *P* < .0001). Variables associated with compliance success on univariate analysis included age (*P* = .0001), gender (*P* = .05), insurance (*P* = .005), previous treatment (*P* = .018) and treatment regimen (*P* = .019) (Table 2). After adjustment by other covariates, clinic setting still had significant effect on compliance (*P* = .0008), and age, gender, and treatment regimen were retained in the final model. Female gender (OR 1.45, 95% CI: 1.08, 1.94), older age (OR 1.03, 95% CI: 1.01, 1.04) and patients who had sofosbuvir/simeprevir compared to sofosbuvir/ledipasvir had higher odds of compliance success; patients who had elbasvir/grazoprevir or dasabuvir/ombitasvir/paritaprevir/ritonavir had lower odds of compliance success compared to sofosbuvir/ledipasvir (Fig. 2).

With 1:1 matching, 752 patients (376 SHC treated, 376 GHC treated) were included in our analysis. Baseline characteristics in 2 groups were balanced (Table 3). A total of 608 (80.9%) patients achieved SVR (316 in the SHC and 292 in the GHC), and 616 (81.9%) patients had compliance success (320 in the SHC and 296 in the GHC). There was significant clinic setting effect on treatment success (*P* = .027), as patients treated in SHC had higher odds of achieving treatment success than those treated in the GHC (OR 1.52, 95% CI: 1.05, 2.12). Clinic setting also had significant effect on compliance success (*P* = .024), as patients treated in SHC had higher odds of achieving compliance success than those treated in the GHC (OR 1.54, 95% CI: 1.06, 2.25).

**Table 3:**
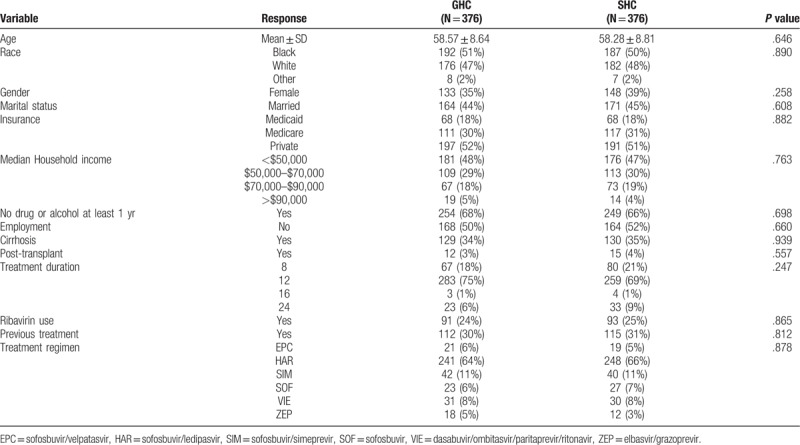
Baseline characteristics differences in SHC and GHC for 1:1 matched cohort using propensity scores.

## 4 Discussion

In a large, diverse, real-world cohort of HCV patients, we found that SVR rates were lower than those reported in registration trials when SVR rates were determined by ITT analysis where patients lost to follow-up were considered as treatment failures. Moreover, we also found that patients who were treated in a clinic with HCV treatment-specific protocols in place were more likely to not only complete treatment and get necessary blood tests to assess, but also to achieve SVR. Those who were compliant with the treatment protocol achieved SVR rates similar to those reported in registration trials, suggesting that the lower SVR reflects non-compliance and not efficacy.

There were significant differences in the clinical characteristics between those who were treated in the SHC and GHC. While those who were treated in SHC had many potential favorable characteristics for a better outcome such as higher income, better insurance, lower unemployment rates or alcohol/drug abstinence, there was a higher proportion of previous treatment failures in the SHC group. This was a potential limitation of our study, but we corrected for these differences by conducting a multivariate analysis and propensity score matching analysis. The independent variables associated with better compliance and treatment success rates in our study were older age, female gender, and treatment in the SHC. In addition, treatment regimen was associated with treatment success. Race, marital status, household income, employment status, cirrhosis, post-transplant status, treatment duration, use of ribavirin, and alcohol/drug abstinence for more than 1 year were not predictors of either compliance or treatment success. After adjusting for other variables, insurance was not independently associated with compliance or treatment success. Propensity score matching analysis included 752 patients and confirmed the effect of clinic setting on treatment success and compliance success; patients treated in the SHC had higher odds of compliance and treatment success than patients treated in the GHC.

In this study, we used less strict criteria to define compliance and treatment success. Since SVR4 has been shown to have high concordance with SVR12, we allowed for obtaining either SVR4 or SVR12 labs as part of the criteria for achieving compliance and treatment success.^[20]^ Despite using these relaxed criteria, our overall compliance rate was only 79.9%. Our study corroborates the observations made in a recent retrospective study of 261 genotype 1 patients treated at an academic center in the USA using ledipasvir/sofosbuvir combination. In their study, 7% did not complete treatment and 15% were lost to follow-up. By ITT analysis, SVR rate was 74%, with lower rates (68% vs. 86%) in treatment naïve patients compared to treatment-experienced patients.^[18]^ In our study, treatment experience had significant effect on SVR in univariate and multivariate analysis. An Australian study also found lower SVR rates (80.4%) with DAA (similar to our SVR rates of 80.1%) and lower SVR rates were attributed to a significant number of patients (14.2%) who were lost to follow-up.^[18]^

Although few previous studies evaluating the real-world effectiveness of DAAs had suggested that SVR rates in real life are similar to those found in registration trials, many of these studies did not include patients who were lost to follow-up or lacked post-treatment SVR testing.^[10–15]^ A large VA study showed lower SVR (82.4%) rates when patients who were lost to follow-up were considered as non-responders, but when analyzed by multiple imputations using logistic regression, SVR rate was 90%.^[16]^ In another prospective community-based study, the SVR rate was found to be only 86% in genotype 1a patients treated with sofosbuvir/ledipasvir.^[17]^ Our results also showed 95% SVR rate when only patients who completed follow-up were analyzed, but only 79.9% when patients lost to follow-up were considered as non-responders.

The reasons for non-compliance with DAA have not been adequately studied. When patients were treated with interferon-containing regimens, side effects were one of the main reasons for non-compliance and discontinuation of treatment.^[21]^ Unlike interferon-containing regimens, DAA are well tolerated with discontinuation rates of less than 1% as compared to ∼20% in interferon-based treatment in registration trials. A post-hoc analysis of 13 registration trials had suggested that SVR rates of 82.5% in patients who were non-adherent to sofosbuvir/ledipasvir combination.^[21]^ In that study, duration of treatment was not related to non-adherence, as in our study, and the only side effect that was reported to be higher in the non-adherent group was flu-like symptoms. Although our results showed that treatment regimen was a significant factor in achieving compliance and treatment success, the majority of our study population received sofosbuvir/ledipasvir; thus, we do not believe that this factor is clinically applicable based on our data. In our study, older age was associated with better compliance. Many hepatitis B virus (HBV) studies, including real-life studies, have also shown that younger age was associated with non-adherence to therapy.^[22–24]^ Unlike HBV treatment, HCV treatment is for a defined period of 8 to 12 weeks, and hence it is difficult to extrapolate HBV or HIV treatment experience to HCV patients treated with DAA. We do not know whether non-compliance of our patients was due to relapse of alcoholism or drug use. However, none of our patients were actively using drugs when treatment was initiated, 64% of patients had abstained from drugs or alcohol for more than 1 year, and the compliance and treatment success rates were similar in those who abstained for more than 1 year compared to those who did not. Moreover, people who inject drugs or consume excess alcohol had shown similar adherence rates as non-drug users with interferon or DAA-based HCV treatment.^[25–28]^ It has been suggested that the non-compliance in patients with HCV is perhaps more complex, and a patient-centered approach that addresses social, emotional, and practical concerns may facilitate adherence and completion of treatment.^[29–31]^

In our study, we showed better compliance and treatment success rates when patients were managed in the SHC. Studies in other areas of medicine have shown that a structured approach with specialized outpatient protocols for prevalent, chronic diseases not only increased provider adherence to disease management guidelines, but also positively impacted disease measures, patient outcomes, and healthcare costs.^[32–37]^ The findings from our study have similar implications for HCV patients. Although DAA regimens are widely available (used by gastroenterologists, hepatologists, and primary care physicians) and highly effective, they are expensive and labor intensive including time-consuming insurance authorization process. Thus, a significant proportion of patients who are lost to follow-up during the treatment process represents a major problem with regards to wasting valuable resources, increasing costs, and potentially serious consequences for treatment failure, such as development of cirrhosis, decompensation, and HCC. We believe that optimization of HCV therapy should involve a structured setting where patients are required to pick-up their medication which will provide an opportunity for the treating physician to review a detailed treatment plan including standardized follow-up visits and laboratory monitoring. The pre-treatment clinic visits also provided an opportunity to educate the patients about potential side effects, the importance of compliance for better cure rates, the drug-drug interactions, HBV reactivation risk, and long-term HCC surveillance in those with advanced fibrosis. Additionally, flowsheet documentation or other electronic tools may facilitate patient tracking and outreach.^[37]^ Since the completion of study, we have started tracking and contacting patients who miss the clinic visits or laboratory testing with an intention to reduce the number of patients lost to follow-up and to identify modifiable variables that result in non-compliance.

In conclusion, our study showed that effectiveness of HCV treatment is suboptimal in the real-world due to patient non-compliance. However, compliance and treatment success rates could be improved to some extent by coordinating treatment in a structured HCV clinic (SHC).

## Author contributions

PJT contributed to the conception and design; NC and PJT drafted the manuscript; NC, SB and CC contributed to or the acquisition of data; TZ did the statistical analysis; AM and HY participated in the critical revision for important intellectual content, and all authors approved the final version, and agree to be accountable for all aspects of the work.

**Conceptualization:** Paul J. Thuluvath.

**Data curation:** Navdeep Chehl, Colleen Cook, Sara Brown.

**Formal analysis:** Talan Zhang, Paul J. Thuluvath.

**Methodology:** Talan Zhang.

**Writing – original draft:** Navdeep Chehl.

**Writing – review & editing:** Anurag Maheshwari, Hwan Yoo, Paul J. Thuluvath.
